# The role of the state government, civil society and programmes across sectors in stunting reduction in Chhattisgarh, India, 2006–2016

**DOI:** 10.1136/bmjgh-2019-002274

**Published:** 2020-07-06

**Authors:** Neha Kohli, Phuong H Nguyen, Rasmi Avula, Purnima Menon

**Affiliations:** 1 Geography, University of Florida, Gainesville, Florida, USA; 2 Poverty, Health and Nutrition Division, International Food Policy Research Institute, Washington, District of Columbia, USA

**Keywords:** nutrition, stunting

## Abstract

**Introduction:**

Childhood stunting has declined in India between 2006 and 2016, but not uniformly across all states. Little is known about what helped some states accelerate progress while others did not. Insights on subnational drivers of progress are useful not just for India but for other decentralised policy contexts. Thus, we aimed to identify the factors that contributed to declines in childhood stunting (from 52.9% to 37.6%) between 2006 and 2016 in the state of Chhattisgarh, a subnational success story in stunting reduction in India.

**Methods:**

We examined time trends in determinants of stunting using descriptive and regression decomposition analysis of National Family Health Survey data from 2005 to 2006 and 2015–2016. We reviewed nutrition-relevant policies and programmes associated with the drivers of change to construct a policy timeline. Finally, we interviewed multiple stakeholders in the state to understand the changes in the drivers of undernutrition.

**Results:**

The regression decomposition analysis shows that multiple factors explain 66% of the change in stunting between 2006 and 2016. Improvements in three key drivers—health and nutrition services, household assets, and sanitation and hygiene—explained 47% of the change in stunting. A shared vision for impact, political stability and capable bureaucracy, state-level innovations, support from development partners and civil society, and community mobilisation were found to contribute to improvements in programmes for health, poverty and sanitation.

**Conclusion:**

Change in multiple sectors is important for stunting reduction and can be achieved in subnational contexts. More work lies ahead to close gaps in various determinants of stunting.

**Key questions**

**What is already known?**

- National success cases in stunting reduction have been documented in the literature but few studies have examined what enables accelerated change in subnational contexts.
- Between 2006 and 2016, India has made progress in reducing stunting among children, but the progress was variable across the states, thus offering an opportunity to learn about subnational successes.

**What are the new findings?**

- Improvements in three areas—health and nutrition services, household socioeconomic status and sanitation—contributed to declines in stunting in Chhattisgarh.
- Changes within programmes and sectors were supported by an overall political environment, bureaucratic capabilities and support from a wide range of stakeholders including civil society.

**What do the new findings imply?**

- Achieving reductions in stunting requires actions in both health and non-health sectors.
- Deliberate efforts to engage a wide range of stakeholders can help to create opportunities to implement effective solutions in diverse sectors, that together create better conditions for stunting reduction.
- Despite a common national framework, a range of factors—politics, administrative capability, community engagement and more—at subnational levels matters tremendously in supporting implementation of solutions for undernutrition.

## Introduction

In the last decade, there has been marked increase in global attention and political momentum to address child malnutrition. The World Health Assembly has set a target of 40% reduction in stunting in children by 2025, which was endorsed by the WHO’s member states. While such global impetus pushes countries to action, blueprints for effective action remain a challenge.

Globally, studies exist of how countries achieved stunting reduction. For instance, there are success stories of national-level stunting reduction from Guatemala, Bolivia, Brazil, Nepal and Bangladesh where policy actions were driven by the need to eliminate hunger.^1 2^ However, there is little understanding of factors that contribute to improvements at a subnational level; such case studies are essential as many countries today operate in decentralised political and administrative set-ups. Expanding research on subnational stunting reduction can help deliver insights on the interplay between national policy and programme frameworks and tailored actions at a subnational level.

India is one of the largest countries in the world, comprising one-sixth of the world’s population. Although India has made progress in reducing stunting among children between 2006 and 2016, the progress has been variable across the states^3^ and within states.^4 5^ The interstate differences in stunting reduction occurred in the context of common national policy framework for programmes including health and nutrition, food security and sanitation. Previous studies have found interstate differences in poverty reduction^6^ and nutritional outcomes^7^ across India to be correlated with political regimes. To our knowledge, there are only two subnational studies of drivers of change in childhood stunting in India: Maharashtra and Odisha.^8 9^ Both these studies showed that change was possible under enabling national programmes and policies targeting health, food security and poverty, along with effective implementation of health and nutrition interventions at the state level.^8–10^

Chhattisgarh, a state in eastern India, was carved out of the state of Madhya Pradesh in 2000 with a population of ~30 million. The decline in stunting in Chhattisgarh between 2006 and 2016 (from 52.9% to 37.6%) was higher than in any other state in India during that period, despite having higher levels of poverty compared with several states.^11^ We sought to understand how the state achieved such remarkable improvements in child stunting in this short period. We aimed to (1) examine the empirical drivers of change in stunting in children in Chhattisgarh; and (2) identify programmatic, social and political factors that contributed to changes in these drivers.

## Methods

We used mixed methods for the study. First, we examined the data on stunting reduction and changes in known drivers of undernutrition descriptively. We then used regression-decomposition analysis to examine the contributions of changes in known determinants of stunting between 2006 and 2016. Second, based on the results of the decomposition analysis, we conducted a literature review and policy analysis to identify the nutrition-relevant policies and programmes that were most likely associated with the drivers of change; we used this review to construct a timeline of policy evolution. Third, we interviewed stakeholders in the state to understand their perceptions about the potential reasons for changes in key programmes and policies. Finally, we integrated insights from all these research methods in drawing together our interpretation of what drove change and what contributed to that change in Chhattisgarh.

### Empirical analysis of publicly available data

We used data from National Family Health Survey 2015–2016 (NFHS-4) and the 2005–2006 (NFHS-3) for Chhattisgarh (n=7971 and 1426, respectively). These data are representative at the state level and provide comprehensive information on child growth and several related determinants. The nutrition outcomes used in this analysis were child height-for-age z-scores (HAZ) and stunting (defined as HAZ<-2 SD).^12^ All analyses used data for the youngest child under 5 years.

We selected a set of immediate and underlying determinants, and interventions based on the UNICEF/Lancet conceptual framework.^13^ The immediate determinants included maternal underweight (body mass index (BMI) <18.5) and child diet. The underlying determinants are maternal education, household socialeconomic status (SES), religion, caste status, sanitation, and village level sanitation and electrification (table 1). Household SES was constructed using a principal component extracted from multiple variables including household ownership of 15 assets. We included the coverage indicators for interventions during pregnancy, at birth and during early childhood. Interventions pertaining to food supplementation were not included in the analysis as there is evidence that beneficiaries self-select into the programme in ways that reverse the association between use of the programme and nutrition outcomes.^14^

**Table 1:** Changes in immediate and underlying determinants of child growth in Chhattisgarh between 2006 and 2016

	2006 Percent/mean	2016 Percent/mean	Change (percentage points)	P value
Immediate determinants				
Maternal height	151.77	151.42	−0.34	0.155
Maternal weight	43.85	47.36	3.51	<0.001
Maternal low BMI (<18.5)	45.11	25.65	−19.46	<0.001
Vegetarian	14.59	13.39	−1.2	0.562
Underlying determinants				
Household level				
Household size, *n*	7.05	6.04	−1.01	<0.001
Having health insurance	2.50	65.53	63.03	<0.001
SES index (0–10), score*	4.33	6.04	1.71	<0.001
Schedule caste	14.10	14.36	0.25	0.913
Schedule tribe	32.11	32.20	0.10	0.981
Other backward classes	45.93	45.77	−0.16	0.966
Other	7.86	7.67	−0.19	0.910
Hindu religion	96.12	95.25	−0.87	0.501
Muslim religion	2.60	2.75	0.15	0.895
Hygiene and sanitation				
Having toilet in household	15.12	41.04	25.92	<0.001
Improved latrine	12.22	32.06	19.84	<0.001
Stool safe disposal	10.15	24.49	14.33	<0.001
Improved drinking water	77.76	90.15	12.39	<0.001
Having pumping water	6.98	14.97	7.99	<0.001
Maternal level				
≤9 years of schooling	88.27	76.10	−12.17	<0.001
High school (10–12)	6.81	15.23	8.42	<0.001
College or higher	4.92	8.67	3.75	0.005
Married before 18	67.08	36.78	−30.3	<0.001
Village factors				
% households having toilet	15.12	41.04	25.92	<0.001
% households having electricity	73.86	96.23	22.37	<0.001
Rural	81.72	77.63	−4.09	0.305
Child				
Boys, *%*	52.44	53.78	1.34	0.480
Age, months	28.54	29.08	0.54	0.331
Birth order	2.98	2.29	−0.69	<0.001
N	935	5223		

Values are means or percentage.

*The socioeconomic status (SES) index was constructed using a principal component extracted from multiple variables including household ownership of 15 assets (car, motorbike, bicycle, television, computer, refrigerator, mobile phone, watch, fan, bed, mattress, table, chair, press cooker, sewing machine), livestock (cow, goat, chicken), house and land, as well as key housing characteristics (housing materials for floor, roof, wall and source of cooking). The first component derived from the component scores was scaled with the range 0–10 to obtain a measure of household wealth relative to other households, with a higher score indicating higher wealth.

ANC, antenatal care; BMI, body mass index; IFA, iron and folic acid; SES, social economic status.

Analytically, we first compared the changes in HAZ, stunting and drivers from 2006 to 2016 by using regression models, adjusting SE for the survey sampling design and applying sampling weights. We then performed regression-decomposition analysis to assess how much the change in each determinant contributed to changes in stunting. This analysis combines the analysis of differences in means of the explanatory variables (X) between 2006 and 2016 and regression estimates of the coefficients associated with these variables (Β_X_) from a pooled regression model. For example, if a determinant has a large regression coefficient (‘marginal effect’) and a large change in its mean over time, then this determinant will play a large role in explaining stunting reduction over time. This method has been used in previous studies to examine changes in undernutrition in other countries.^2 15 16^ Based on the results of the decomposition analysis, we identified areas to focus for the policy analysis.

### Literature review and policy analyses

The literature review had two objectives: first, to construct a policy timeline and analyse policy changes over the period of stunting reduction; and second, to gather additional literature to support overall analysis and interpretation. We reviewed published and grey literature, government documents and websites to construct a timeline of programme and policy implementation in the state between 2000 and 2017. The websites reviewed include that of the Departments of Health and Family Welfare, Women and Child Development, and Food Civil Supplies and Consumer Protection of the state of Chhattisgarh. To identify relevant literature, we searched *Google Scholar* and the archives of the Indian journal *The Economic and Political Weekly*. We focused our search on institutional mechanisms, health, nutrition and food security programmes, and on programmes related to stunting determinants identified in the decomposition analysis.

### Stakeholder interviews

We conducted interviews with key stakeholders (n=17) at the state level between November 2017 and January 2018 to understand factors associated with changes in nutrition-relevant policies and programmes. We used a snowball sampling method^17^ to identify interviewees. We first identified two key informants who were knowledgeable in health and nutrition programmes in the state. These key informants then provided a first list of potential interviewees, who then identified other interviewees. The interviewees from the government were identified such that their time in service corresponded with the time when changes in stunting were observed. At the time of the interview, not all interviewees were working in the state but had worked there in the preceding years. Overall, we interviewed government officials (n=7) and stakeholders from civil society/non-government organisations (NGO) (n=6), development partners (n=2) and academia (n=2).

Semistructured interview guides were tailored to specific stakeholder type using insights from the key informant interviews and the policy timeline. In addition to specific questions about programmes, which varied by the knowledge and experience of stakeholders, the interview guides included key questions about drivers of change in nutrition-relevant programmes and about the roles of different stakeholders in the change process (online supplementary table 1A).

10.1136/bmjgh-2019-002274.supp1 Supplementary data

All interviews were audio-recorded after seeking informed consent from the participants. Confidentiality was ensured prior to beginning any interview. The transcripts were coded in *Excel* using a code list based on the interview guide, allowing for emergent codes (online supplementary table 1B). The codes were then clustered into broad thematic areas, which were later summarised into select elements of a conceptual framework for the drivers of programmatic changes (online supplementary table 1C).^18^

### Patient and public involvement statement

As our study used a national-level secondary dataset, the study participants were not involved in the design of the study. The stakeholders who were interviewed for the study were not involved in the study design or development of the research questions. We shared initial analytical findings with stakeholders including government officials, development partners, civil society members and academicians for their feedback, which was then used for data triangulation and for identifying areas to explore further.

## Results

### Changes in linear growth and stunting between 2006 and 2016

**Figure 1:**
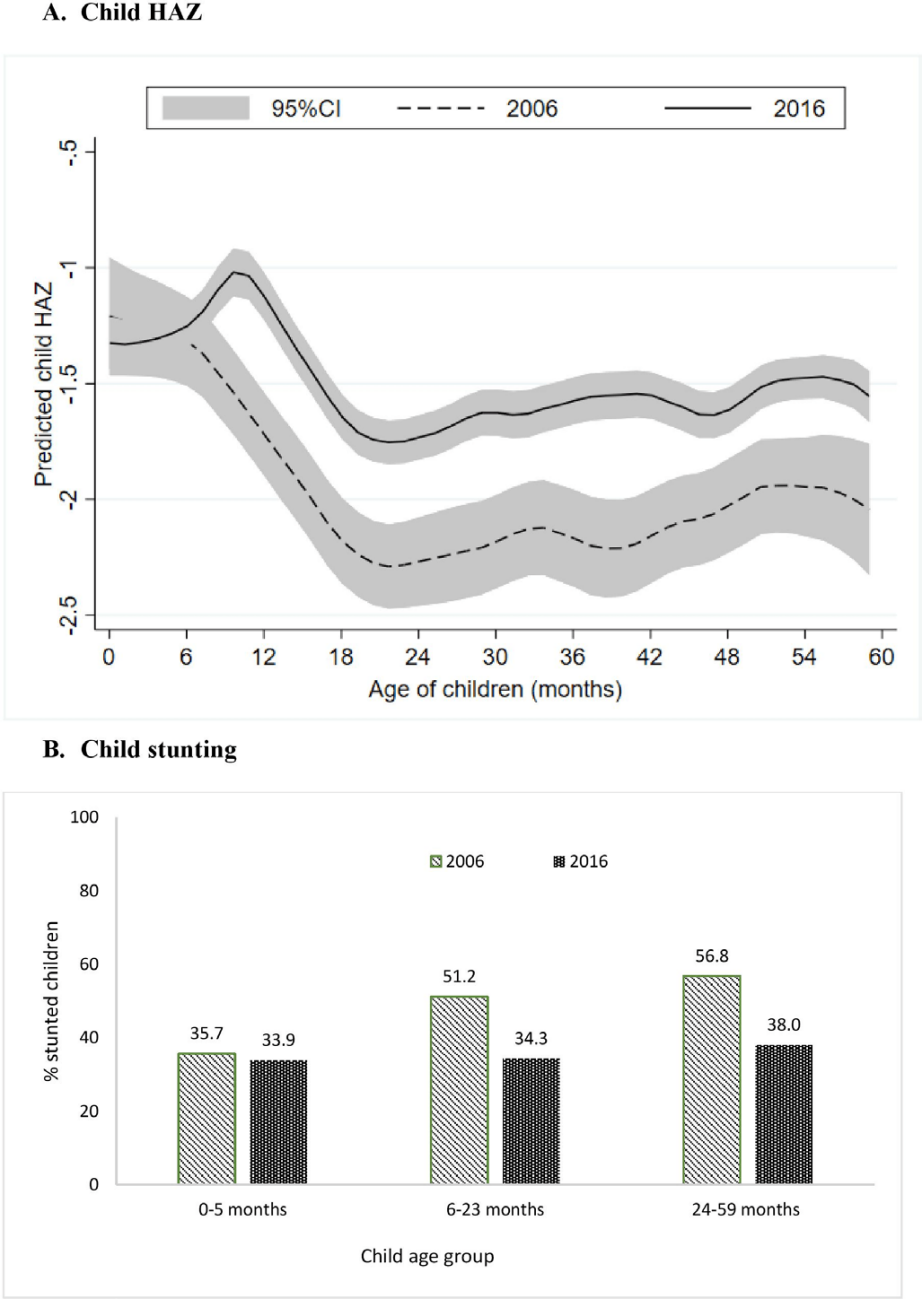
Changes in height-for-age z-score (HAZ) and stunting between 2006 and 2016 for different age groups of children in Chhattisgarh. (A) Child HAZ. (B) Child stunting.

Between 2006 and 2016, stunting among children <5 years of age in Chhattisgarh declined from 51.6% to 35.9% and HAZ improved from −1.96 to −1.44 SD. However, there was no difference in HAZ at birth (~−1.3 SD) and in the first 6 months of age (figure 1A). HAZ scores for children 6–59 months improved by about 0.52 SD over the decade, with slightly larger improvements among children >20 months of age. Stunting affected a third of children aged 0–5 months, with marginal change between the two time periods (figure 1B). There were, however, substantial improvements in the proportion of children stunted between 6 and 59 months (~18 pp). Since the change was minimal for children <6 months, we focused our analysis of drivers of change on children of 6–59 months of age.

### Changes in immediate and underlying determinants of child growth and in the reach of interventions between 2006 and 2016

Between 2006 and 2016, several known determinants of stunting improved significantly in Chhattisgarh (table 1). The proportion of women with low BMI (<18.5) declined from 45.1% to 25.7%. At the household level, SES, hygiene and sanitation and early marriage in girls improved.

**Figure 2:**
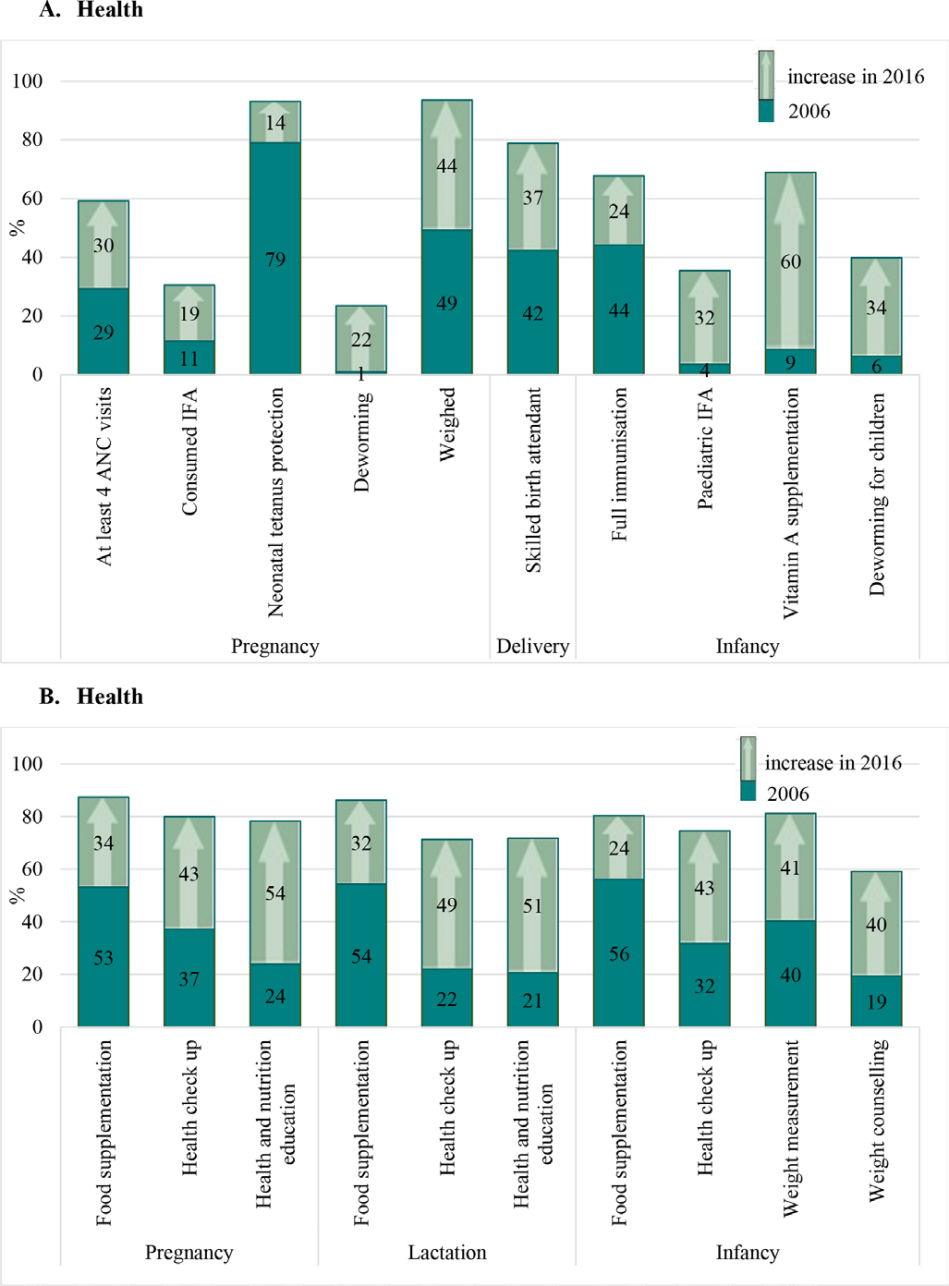
Changes in health and nutrition intervention coverage in Chhattisgarh between 2006 and 2016. (A) Interventions delivered through the health system. (B) interventions delivered through the Integrated Child Development Services (ICDS). ANC, antenatal care; IFA, iron and folic acid.

The coverage of all health and nutrition interventions substantially improved (figure 2A). These include care during pregnancy (antenatal care (ANC): 29%–59%, iron folic acid (IFA) consumption: 11%–30%, neonatal tetanus protection: 79%–93%, and deworming: 1%–24%), care during delivery (skilled birth attendant: 42%–79%) and nutrition interventions focused on children (full immunisation: 44%–68%, paediatric IFA: 4%–36%, vitamin A supplementation: 9%–69% and deworming: 6%–40%). Coverage of food supplementation during pregnancy, lactation and early childhood increased phenomenally from 53% to 87%, 54% to 86%, and 56% to 80%, respectively, and weighing of children improved from 40% to 81% (figure 2B). Interdistrict variability was observed in the coverage of only some interventions in 2016, particularly during pregnancy (ANC, IFA) and infancy (deworming) (table 2). Use of ANC ranged from a low of 34.6% in one district in Chhattisgarh to a high of 76.3% in another district and coverage of deworming ranged between for children ranged between 23.4% and 64.1%. Coverage of food supplementation was high with negligible interdistrict variability during pregnancy and infancy.

**Table 2:** Coverage of health and nutrition interventions delivered through the health system and the Integrated Child Development Services (ICDS) programme in 2016, by district, in Chhattisgarh

	<25%		25-<50%		50-<75%		>75%													
		Chhatt isgarh	Bas tar	Bija pur	Bilas pur	Dakshin Bastar Dante wada	Dhamtari	Durg	Janjgir Champa	Jash pur	Kabir dham	Korba	Koriya	Maha samund	Nara yanpur	Rai garh	Rai pur	Rajnan dgaon	Sur guja	Uttar Bastar Kanker
**Interventions delivered through the health system**																				
**Pregnancy**	≥4 ANC	**59.1**	**55.7**	**49.1**	**60.7**	**60.5**	**76.3**	**59.9**	**44.3**	**34.6**	**43.8**	**52.9**	**45.1**	**60.8**	**40.9**	**67.6**	**76.2**	**64.2**	**41.7**	**72.1**
	Received IFA/tab /syrup	**91.4**	**90.4**	**86.3**	**93.3**	**85.8**	**93.7**	**92.4**	**86.5**	**89.3**	**92.8**	**93.8**	**90.8**	**92.8**	**78.9**	**93.3**	**92.0**	**93.5**	**86.9**	**92.7**
	Consume 100+IFA	**30.6**	**29.2**	**21.3**	**39.4**	**26.2**	**43.0**	**37.6**	**18.5**	**26.1**	**19.1**	**24.3**	**39.6**	**23.8**	**23.7**	**21.3**	**31.6**	**40.0**	**18.5**	**38.5**
	Neonatal tetanus protection	**92.9**	**95.9**	**94.4**	**96.5**	**93.5**	**98.9**	**95.0**	**91.2**	**90.8**	**91.9**	**91.1**	**93.3**	**95.4**	**84.7**	**92.2**	**91.7**	**95.8**	**83.4**	**91.9**
	Deworming	**23.9**	**17.8**	**28.7**	**39.1**	**13.7**	**18.7**	**22.5**	**15.5**	**13.8**	**17.0**	**24.4**	**27.8**	**24.9**	**23.4**	**15.3**	**17.9**	**35.2**	**24.7**	**17.2**
	Weighing	**93.9**	**97.3**	**99.0**	**98.4**	**93.3**	**99.6**	**98.2**	**89.2**	**87.3**	**86.1**	**93.3**	**88.4**	**99.4**	**90.4**	**91.6**	**94.4**	**99.3**	**79.7**	**94.3**
**Delivery**	Skill birth attendant	**80.2**	**77.1**	**82.9**	**87.7**	**70.8**	**91.4**	**84.1**	**69.2**	**68.3**	**59.3**	**75.2**	**76.8**	**85.9**	**75.2**	**79.6**	**81.1**	**93.2**	**69.4**	**85.8**
**Infancy**	Full immu nisation	**76.4**	**70.9**	**85.7**	**80.9**	**67.0**	**89.1**	**91.1**	**71.1**	**52.3**	**61.7**	**81.0**	**73.9**	**74.1**	**61.5**	**67.1**	**80.2**	**86.5**	**65.7**	**81.0**
	Paediatric IFA	**35.5**	**32.2**	**41.2**	**47.8**	**31.1**	**34.9**	**29.0**	**19.1**	**31.9**	**36.1**	**31.0**	**45.2**	**26.2**	**42.1**	**36.4**	**39.5**	**42.5**	**33.9**	**33.6**
	Vitamin A supple mentation	**68.9**	**70.1**	**77.6**	**77.5**	**66.6**	**61.5**	**66.9**	**63.9**	**63.2**	**72.9**	**62.1**	**72.9**	**60.4**	**70.5**	**77.7**	**70.8**	**76.9**	**59.1**	**68.2**
	Deworming	**39.9**	**35.5**	**61.1**	**64.1**	**36.5**	**35.8**	**41.4**	**24.3**	**23.4**	**31.1**	**38.9**	**45.8**	**34.8**	**55.9**	**35.2**	**32.8**	**60.2**	**31.6**	**38.6**
	Food supple mentation	**84.7**	**89.4**	**82.8**	**87.0**	**87.7**	**90.1**	**81.2**	**85.5**	**84.5**	**90.9**	**81.3**	**78.6**	**88.2**	**81.4**	**81.5**	**80.8**	**87.4**	**85.6**	**87.9**
	Health & nutrition education	**71.1**	**78.5**	**76.9**	**77.7**	**77.5**	**83.2**	**75.8**	**71.0**	**68.8**	**68.1**	**69.4**	**62.0**	**77.0**	**66.3**	**55.7**	**63.1**	**75.6**	**64.5**	**75.5**
	Full immunisation	**76.4**	**70.9**	**85.7**	**80.9**	**67.0**	**89.1**	**91.1**	**71.1**	**52.3**	**61.7**	**81.0**	**73.9**	**74.1**	**61.5**	**67.1**	**80.2**	**86.5**	**65.7**	**81.0**
	Vitamin A	**68.9**	**70.1**	**77.6**	**77.5**	**66.6**	**61.5**	**66.9**	**63.9**	**63.2**	**72.9**	**62.1**	**72.9**	**60.4**	**70.5**	**77.7**	**70.8**	**76.9**	**59.1**	**68.2**
	Paediatric IFA	**35.5**	**32.2**	**41.2**	**47.8**	**31.1**	**34.9**	**29.0**	**19.1**	**31.9**	**36.1**	**31.0**	**45.2**	**26.2**	**42.1**	**36.4**	**39.5**	**42.5**	**33.9**	**33.6**
	Deworming for children	**39.9**	**35.5**	**61.1**	**64.1**	**36.5**	**35.8**	**41.4**	**24.3**	**23.4**	**31.1**	**38.9**	**45.8**	**34.8**	**55.9**	**35.2**	**32.8**	**60.2**	**31.6**	**38.6**
**Interventions delivered through the Integrated Child Developmnt System**																				
**Pregnancy**	Food supple mentation	**87.2**	**93.3**	**89.7**	**88.7**	**88.0**	**94.3**	**82.0**	**84.2**	**89.0**	**93.1**	**83.2**	**84.3**	**94.2**	**84.8**	**87.2**	**83.0**	**91.9**	**86.2**	**90.8**
	Health & nutrition education	**78.4**	**85.0**	**87.4**	**81.5**	**81.1**	**90.7**	**77.6**	**70.1**	**71.3**	**82.4**	**72.2**	**66.9**	**86.2**	**77.6**	**74.4**	**76.9**	**85.3**	**70.5**	**87.8**
**Lactation**	Food supple mentation	**84.7**	**89.4**	**82.8**	**87.0**	**87.7**	**90.1**	**81.2**	**85.5**	**84.5**	**90.9**	**81.3**	**78.6**	**88.2**	**81.4**	**81.5**	**80.8**	**87.4**	**85.6**	**87.9**
	Health & nutrition education	**71.1**	**78.5**	**76.9**	**77.7**	**77.5**	**83.2**	**75.8**	**71.0**	**68.8**	**68.1**	**69.4**	**62.0**	**77.0**	**66.3**	**55.7**	**63.1**	**75.6**	**64.5**	**75.5**
**Infancy**	Food supple mentation (6–35 months)	**88.0**	**89.8**	**86.1**	**92.5**	**84.0**	**92.3**	**81.5**	**93.1**	**89.6**	**93.7**	**83.1**	**83.8**	**88.3**	**85.2**	**89.8**	**82.2**	**92.5**	**91.8**	**93.2**
	Weighing	**79.0**	**83.3**	**84.2**	**84.8**	**77.6**	**81.3**	**75.3**	**72.4**	**79.1**	**79.3**	**76.2**	**73.4**	**78.9**	**73.9**	**78.9**	**74.6**	**85.7**	**78.7**	**85.1**
	Counsel on child growth after weighing	**57.7**	**66.2**	**65.6**	**66.5**	**44.0**	**71.1**	**52.5**	**51.6**	**49.3**	**55.3**	**49.4**	**51.4**	**65.8**	**57.0**	**57.3**	**53.7**	**64.9**	**48.9**	**75.6**

Data source: National Family Health Survey 2015–2016. Rows represent the interventions and the columns represent coverage data at the state level and within each district in Chhattisgarh. The colours indicate the extent of coverage in each district (eg, the lowest level of coverage is shaded in red and the highest level of coverage is shaded in blue) and provide information on interdistrict variability.

ANC, antenatal care; ICDS, Integrated Child Development Services; IFA, iron and folic acid.

### Contribution of changes in determinants and intervention coverage to changes in stunting

Drawing on insights from the descriptive analysis in the trends in determinants, we conducted multivariate regression analyses to assess associations between key determinants and child stunting. Significant associations were found between low maternal BMI, living in rural area or in a low SES household, or belonging to disadvantaged caste with higher odds of stunting (table 3). In contrast, higher maternal education, access to health and nutrition services, higher hygiene and sanitation score at household or village levels were associated with lower odds of stunting. We found similar results for children <6 months (online supplementary table 2). Results of these regression analyses using 2006 data and 2016 data separately or as pooled data were similar. Therefore, we used findings from pooled data–based analysis to derive regression coefficients for the decomposition analyses.

**Table 3:** Associations between selected factors and stunting among children 6–59 months in India

	2006		2016		Pooled	
	β	95% CI	β	95% CI	β	95% CI
Immediate determinants						
Maternal low BMI (<18.5)	0.03***	0.02 to 0.05	0.05***	0.04 to 0.05	0.04***	0.04 to 0.05
Vegetarian	0.02	−0.00 to 0.03	0.02***	0.01 to 0.03	0.02***	0.01 to 0.03
Health and nutrition services						
Health and nutrition services	−0.02***	−0.03 to −0.02	−0.01***	−0.01 to −0.01	−0.01***	−0.01 to −0.01
Underlying determinants						
Household level						
Household size	0	−0.00 to 0.01	0.00***	0.00 to 0.01	0.00***	0.00 to 0.01
SES quintile 1	0.12***	0.09 to 0.16	0.10***	0.08 to 0.11	0.10***	0.09 to 0.12
SES quintile 2	0.09***	0.05 to 0.12	0.07***	0.06 to 0.09	0.07***	0.06 to 0.08
SES quintile 3	0.07***	0.04 to 0.10	0.05***	0.03 to 0.06	0.05***	0.04 to 0.06
SES quintile 4	0.04*	0.01 to 0.07	0.03***	0.02 to 0.04	0.03***	0.02 to 0.04
Any household member has health insurance	0	−0.04 to 0.04	−0.02***	−0.02 to −0.01	−0.01***	−0.02 to −0.01
Scheduled castes	0.06***	0.03 to 0.08	0.06***	0.05 to 0.07	0.06***	0.05 to 0.07
Scheduled tribe	0.04*	0.01 to 0.07	0.04***	0.02 to 0.05	0.04***	0.02 to 0.05
Other backward classes	0.02*	0.00 to 0.04	0.03***	0.02 to 0.04	0.03***	0.02 to 0.04
Hindu religion	−0.01	−0.04 to 0.02	0.01	−0.01 to 0.02	0	−0.01 to 0.02
Muslim religion	0.01	−0.02 to 0.05	0.04***	0.02 to 0.06	0.04***	0.02 to 0.05
Hygiene and sanitation						
Hygiene score	−0.01**	−0.02 to −0.00	−0.02***	−0.02 to −0.01	−0.02***	−0.02 to −0.01
Maternal level						
High school (10-12)	−0.07***	−0.10 to −0.05	−0.05***	−0.06 to −0.04	−0.05***	−0.06 to −0.05
College or higher	−0.10***	−0.13 to −0.07	−0.08***	−0.09 to −0.07	−0.08***	−0.10 to −0.07
Married before 18	0.01	−0.01 to 0.02	0	−0.00 to 0.01	0.01	−0.00 to 0.01
Village factors						
% households having toilet	−0.05*	−0.08 to −0.01	−0.04***	−0.05 to −0.02	−0.04***	−0.05 to −0.02
% households having electricity	−0.02	−0.05 to 0.01	−0.05***	−0.06 to −0.03	−0.04***	−0.06 to −0.03
Rural	−0.00	−0.03 to 0.02	0.02**	0.00 to 0.03	0.01*	0.00 to 0.02
Child						
Birth order	0.01*	0.00 to 0.01	0.01***	0.01 to 0.02	0.01***	0.01 to 0.01
Boy	0.01	−0.00 to 0.02	0.02***	0.01 to 0.03	0.02***	0.01 to 0.02

*p<0.05; ^**^p<0.01; ^***^p<0.001.

ANC, antenatal care; BMI, body mass index; IFA, iron and folic acid; SES, social economic status.

**Figure 3:**
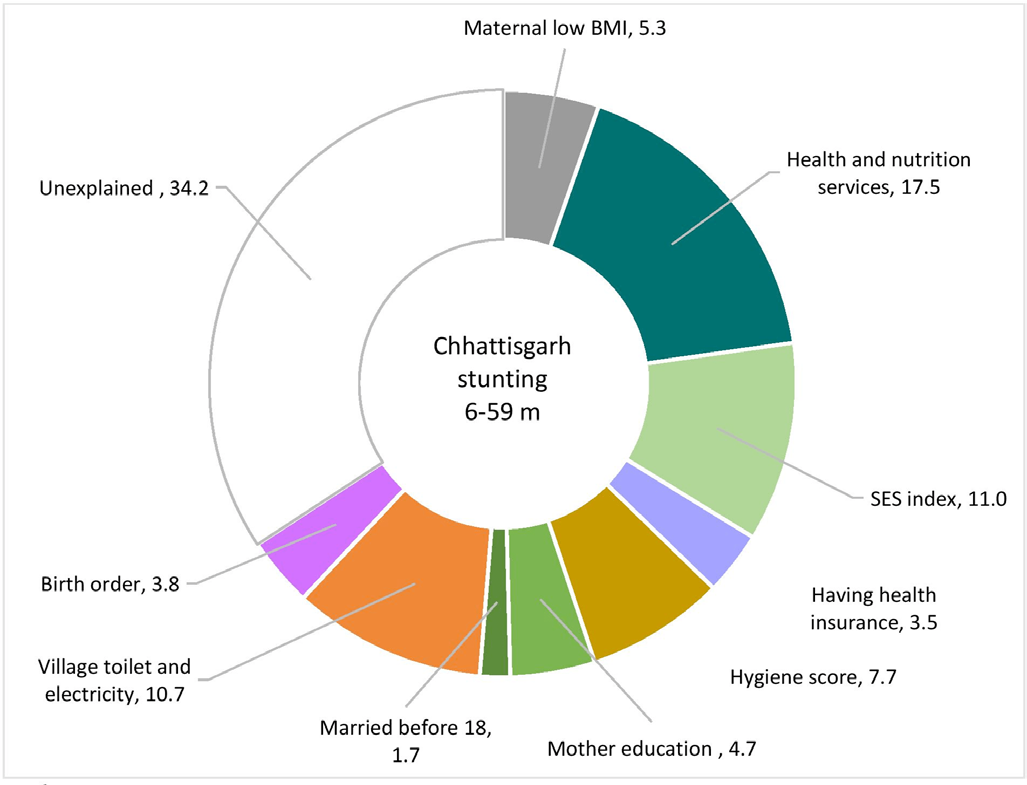
Factors contributing to the changes in stunting in Chhattisgarh between 2006 and 2016 (values are percent shares of the change).

We combined the coefficients selected in the final pooled regression models (table 3) with the changes in determinants from table 1 and figure 2 to examine which factors significantly explained improvement in stunting among children 6–59 months in Chhattisgarh. Our analysis indicates that improvement in health and nutrition services accounts for 17.5% of actual changes in stunting, followed by improvements in SES (11%), village sanitation and electrification (11%), household hygiene (7.7%), maternal BMI (5.3%), maternal education (4.7%) and having health insurance (3.5%) (figure 3). All these factors together explained 66% of the actual change in stunting or 81% change in HAZ (online supplementary figure 1).

10.1136/bmjgh-2019-002274.supp2 Supplementary data

Based on these decomposition analysis results, we selected the priority areas for the policy analysis and stakeholder interviews to understand what factors may have contributed to (1) advancements in nutrition and health services; (2) improvements in SES at the household level and in maternal BMI (which is also related to improvements in economic conditions)^19^; and (3) improvements in sanitation.

#### Advancements in nutrition and health services

**Figure 4:**
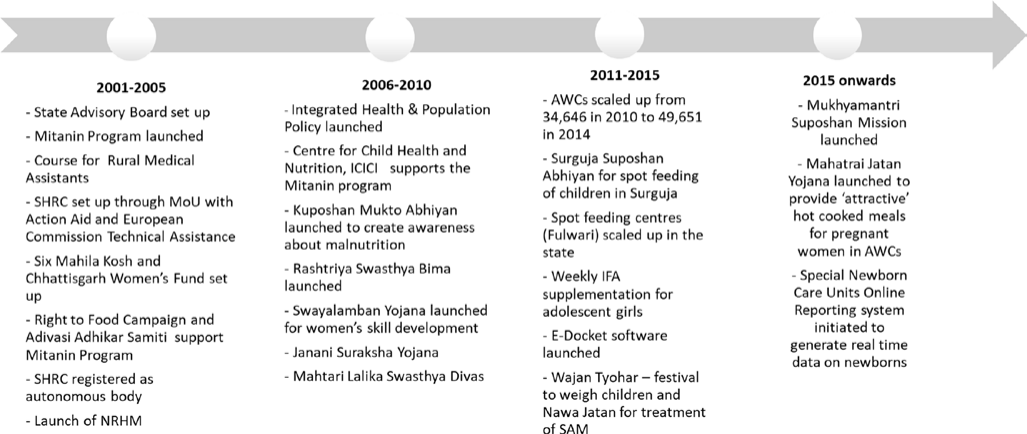
Policy timeline of the health and nutrition programmes. AWC, Anganwadi Center; IFA, iron and folic acid; NRHM, National Rural Health Mission; MOU, Memorandum of Understanding; SAM, Severe Acute Malnutrition; SHRC, State Health Resource Center.

Improvements in the coverage of interventions could be attributed to the gradual programme evolution (figure 4), increase of resources and strengthening of outreach through innovations, often involving the community. There was an expansion of health and nutrition services through national and state-level reforms. The state attempted quite early—in 2001—to build technical capacity in its human resources for health by initiating a certified medical course to train Rural Medical Assistants to serve in remote areas of Chhattisgarh.^20^ In 2002, the state government launched a massive community health worker programme*—Mitanin (translates to friend*), wherein women volunteers provided family-level outreach services, facilitated social mobilisation to improve health system and their activities evolved to focus on child survival and essential care of newborn babies.^21^ The *National Rural Health Mission (NRHM)*, which was launched in 2005 to provide quality health services to rural poor with a specific focus on maternal and child health, provided additional resources to states. Under the mission, ANC was expanded and included critical preventive contacts for nutrition. The new worker introduced as part of NRHM was based on the *Mitanin* model.

During the same time, a national-level impetus led to universalisation of India’s flagship programme*—Integrated Child Development Services* (*ICDS)*, which provides health and nutrition services to pregnant and lactating women, children below 6 years, and adolescent girls through a network of village-level centres. This facilitated expansion of food supplementation, growth monitoring, and health and nutrition education interventions.

*How did these programmatic changes occur in the state?* Stakeholders noted multiple contributions to building these health and nutrition programmes. For instance, the State Health Resource Centre, which provided technical assistance for the implementation of *Mitanin* programme, received funding first from the European Commission and later from the *NRHM*.^22^ A day-care-cum-spot-feeding programme for children 6 months to 3 years in tribal areas was conceptualised at the district level in 2012 to address malnutrition, and was scaled up across the state between 2012 and 2014.^23^ Other state innovations included an annual campaign to promote weighing of children and a programme for the treatment of severely malnourished children.^24^

The stakeholders indicated bureaucratic leadership and capability at state and district levels to be associated with various efforts to improve the reach and quality of health and nutrition programmes. One government official said he was tasked with the ‘primary responsibility’ to ‘figure out a way to reach far-flung tribal areas’. He used his prior experience in implementing innovations in another state to implement programmes in Chhattisgarh. Civil society members and development partners had positive opinion about the state bureaucracy and felt that the officials were responsive, enthusiastic and optimistic.

Stakeholders perceived political support and stability in tenure to be an underlying supporting factor for changes in policies and programmes for health and nutrition. “The bureaucrats had a role to play but they were able to do it because the highest powers within the political system were with it,” said a respondent from civil society.

Civil society and NGOs, including several people involved in the Right to Food Campaign, were widely recognised as forces for driving programmatic changes in the state. Grassroots-level action to improve services for child nutrition was initiated by an organisation of *Adivasis* and the Right to Food Campaign.^25^ *Adivasi (translates to original inhabitants*) is widely employed in self-designation by ‘tribal people’ (a more colonial reference) of these parts of India.^26^ One NGO representative while acknowledging the role of civil society in health reforms also perceived that the government was a greater, if not an equal partner in the success of the reforms—“Every single change is because government made it happen.” This camaraderie between the NGO and the state is part of a series of events that occurred in a specific historical context. With the creation of a new state, there was a window of opportunity and an incentive for stakeholders to help demonstrate possibility of progress. “Chhattisgarh was neglected by Madhya Pradesh so now there was a chance to come out of the shadows,” said a respondent from civil society. Another respondent perceived that since the newly formed state did not have inherent capacity, it was open to collaborations to build necessary state capacity. Thus, stakeholders from government and NGOs with experience in the ‘politics of healthcare’ and implementing livelihood programmes came together to conceptualise health reforms in the state. The civil society and the NGO community worked together and informally with the government to improve health sector in the state.

#### Improvements in SES and maternal BMI

Our empirical findings suggest improvements in SES and in women’s BMI contributed to changes in stunting. To understand factors that might have contributed to these improvements, we examined poverty reduction focusing on the state’s approach to economic growth and to social protection via work and food-related safety net programmes.

First, the poverty declines were reflected in improved household SES in our data analysis. We found that the poverty headcount ratio, particularly in rural areas, declined between 2005 and 2012, with a sharp decline between 2010 and 2012 (table 4). Monthly per capita consumer expenditure on food reduced, while non-food expenditures increased. Between 2005 and 2012, the job growth was higher in Chhattisgarh compared with other advanced states, and the rapid job growth was observed in the construction and service sectors,^27^ both of which could have contributed to poverty decline. The progress, however, was not uniform across the state. Livelihood in remote and forested areas remains compromised and a majority of the *Adivasis* and *Dalits (a population in India discriminated against based on caste)* have limited employment opportunities except in unskilled sectors.^28^ Another potential contributor to poverty declines in the state is the National Rural Employment Guarantee Act (NREGA) that guarantees 100 days of wage employment every year to households with adults. Although the number of workdays used in some districts was between 55 days or less, there was a net increase of 23%–160% in household income in those districts in 2008–2009 compared with 2005–2006.^29^ One stakeholder indicated that NREGA was implemented well between 2006 and 2010 as there were social audits.

**Table 4:** Changes in selected characteristics of the dimensions of poverty

	2005	2010	2012
Poverty headcount ratio (%)			
Total	49.4	48.7	39.9
Rural	55.1	56.1	44.6
Urban	28.4	23.8	24.8
Monthly per capita expenditure (in INR)			
All items	519.2	584.0	626.9
Food items	324.6	317.9	207.3
Non-staples	273.6	402.4	447.6
Non-food	194.6	266.1	419.5
Change in wage/salary (in INR)*			
Regular wage/salaried employees†	116.0	202.3	140.6
Casual labourers‡ in public works	61.0	61.8	71.9
Casual labourers‡ in other work	34.0	49.4	48.4
Change in jobs (%)			
Workers in agriculture	77		73
Workers in industry	10		13
Workers in services	13		14
Workers who are self-employed	52		54
Workers who are salaried	8		10
Workers on casual wage	40		36

Poverty headcount ratio (Reserve Bank of India); monthly per capita expenditure (calculated from the Household Consumption Survey of the National Sample Survey Organisation), change in wages and jobs (Calculated from the Employment and Unemployment Survey data of the National Sample Survey Organisation).

*Wage/salary recorded for person in a day.

†15–59 years of age who are casually engaged in other’s farm or non-farm enterprises (both household and non-household) and, in return, received wages according to the terms of the daily or periodic work contract.

‡Persons who work in other’s farm or non-farm enterprises (both household and non-household) and received salary or wages on a regular basis (ie, not on the basis of daily or periodic renewal of work contract). This category includes not only persons getting time wage but also persons receiving piece wage or salary and paid apprentices, both full time and part time.

**Figure 5:**
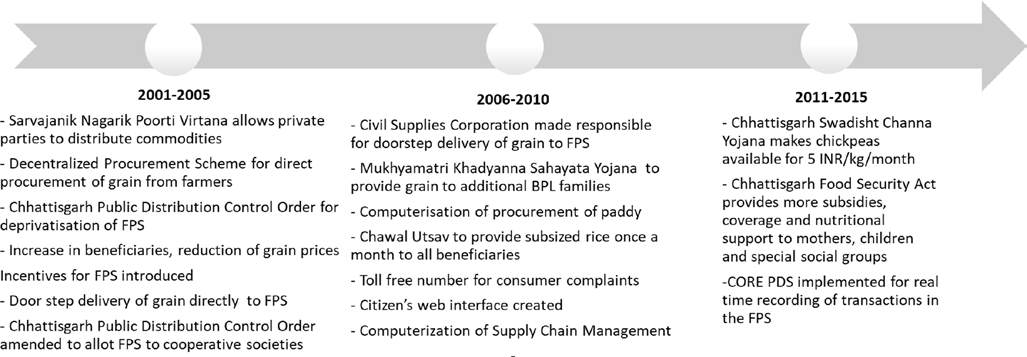
Policy timeline of the Public Distribution System (PDS). FPS, fair price shops.

Finally, both stakeholders and the literature note that the Public Distribution System (PDS), India’s food subsidy programme, helped in poverty reduction.^28^ Between 2005 and 2012, the household uptake of rice from the fair price shops (FPS) of the PDS increased from 21% to 56%, largely due to substantial reforms to the programme. A gradual reform process was undertaken (figure 5) to expand coverage, build efficiency and transparency in procurement and distribution of grains.^30 31^ First, in 2004, the Chhattisgarh Public Distribution (Control) Order deprivatised FPS, transferring them to local bodies.^30^ In 2007, under the chief minister’s leadership, the coverage of households was expanded, reaching nearly 90% of the state’s households, with an intent to achieve political gains.^32^ Additional subsidies for rice were introduced in 2008 along with rapid computerisation of the supply chain management.

Political stability and bureaucratic efficiency and leadership were noted to have enabled programmatic reforms in the PDS. The political party that has been in power for over 10 years continued the PDS reform process that was initiated by the previous government.^33^ Lastly, the Right to Food Campaign, especially, is known to have strengthened the implementation of the programme through building consumer demand and conducting social audits to identify gaps in the system.

Stakeholders reaffirmed the findings in the literature on the well functioning of PDS in the state and attributed it to the reduction in rural poverty. “The Chief Minister was referred to as the ‘rice man,’” said a respondent. It was perceived that the impetus for change originated from the creation of a new state and the political party’s interest to remain in power. “There was a need to project itself better than its poor status under the former state,” said one respondent.

Overall, the combination of the impetus of a new state, job growth related to the massive push for development, investments in the social-safety nets related to work (NREGA) and to food security (PDS), is likely to have contributed to the improvements seen in the household SES.

#### Improvements in sanitation

Between 2006 and 2016, household with access to toilets, households with piped water and safe stool disposal improved (table 1). While the total sanitation campaign began in 2006 in the state, which provided the impetus, the state’s commitment to achieve the sanitation goals led to initiation of innovations. These included engaging women to raise community awareness and ownership, incentivising communities and implementing the UNICEF-supported Community Approach to Total Sanitation (CATS)^34^ to achieve open-defecation-free communities. In 2015, implementation of sanitation programmes was shifted from the Public Health Engineering Department to the Rural Development Department, which facilitated ease of communication and execution of the programmes.^34^

Stakeholders reaffirmed the state’s commitment to sanitation. “It was in 2015 that the government officially shifted its focus from construction to behaviour change,” said a respondent. A few stakeholders echoed that the shift to rural development department facilitated changes. A stakeholder from the development sector said that the departmental shift facilitated change in the approach to addressing the sanitation problem in the state.

Overall, our results indicate that changes in childhood stunting in Chhattisgarh were most visible among children 6–59 months of age. These changes were accompanied by changes in relevant immediate and underlying determinants of child undernutrition, which in turn were preceded by, and supported by, a range of policy and programme actions undertaken by the government along with supporting actions from all of society.

## Discussion

Our study used a range of research methods to identify what factors reduced stunting in Chhattisgarh, what policies and programmes played a role and how these changes occurred. The quantitative research showed that changes in health and nutrition services, household assets, and hygiene and sanitation explain two-thirds of the change in stunting among children <5 years in Chhattisgarh. The policy analysis highlighted that expansion of health and nutrition programmes at the national level contributed to the changes seen in programme coverage. Similarly, state-level innovations to grow the economy and to reduce poverty through work and food security programmes supported poverty decline. And finally, the qualitative interviews with stakeholders identified the range of factors that supported policy changes—these included strong political leadership fuelled partly by the energy of establishing and growing a newly formed state, supported by capable bureaucratic leadership, collaborations among development partners and civil society, and community engagement. Together, these factors contributed to changes in programmes that led to improved immediate and underlying determinants of stunting (table 5).

**Table 5:** Analysis of factors contributing to the policy and programmatic reforms of three major programmes in Chhattisgarh

	Health and ICDS programmes	Public distribution system
**Vision for impact**		
Statehood	New policy energy for change created when the state was carved out from Madhya Pradesh. New state formation enabled opportunities for government and civil society to aid the change process through a vision for impact.	
Development indicators	Poverty and high IMR motivated reforms that supported the goal for impact.	
**Enabling environment**		
Political leadership	Political parties in power until 2018 supported the *Mitanin* programme and PDS in the state since its inception in 2000.	
Political stability	The state political leadership remained the same for three consecutive terms between 2003 and 2018 and continued the policy efforts of the former ruling party.	
Other political factors	Political leaders became interested in the health reforms once the IMR reductions happened in 2003–2004. Beneficiaries of the *Mitanin* seen as major contributors to the vote bank.	Push for reforms in 2007 were spurred by the ruling party’s loss to the opposition in a constituency. PDS beneficiaries seen as major contributors to the vote bank.
Bureaucratic leadership	Several health sector programmes (*Mitanin,* creche programme, an annual child-weighing campaign, and a programme to treat severely malnourished children) received support of bureaucratic leadership at the state and district levels.	Computerisation reforms backed by bureaucratic leadership in the department of food and civil supplies.
Bureaucratic capability	Several health sector schemes associated with an able and experienced bureaucracy. Some functionaries of the reform process had the experience of working with reforms in Madhya Pradesh.	
**Operational capacities**		
Technical	Rural Medical Assistance Scheme brought in more human resources.	Extensive use of technology to make the PDS more efficient and transparent.
Financial	Diverse sources of funding including untied funds, District Mineral Fund (royalty charged by the government for extraction of minerals) and the private sector.	
**Delivering interventions through platforms**		
Policy guidance	The National Rural Health Programme policy framework provided guidance to implementation of health sector reforms. Integrated Child Health Development Services provided guidance to implement nutrition interventions.	
Resources	NRHM enabled financial, technical and infrastructural resources for programme implementation.	Centre supported state-led reforms.
**Champions and catalysts**		
Local NGOs and civil society	State Health Resource Centre seen as a major contributor to health sector reforms. A united NGO force helped to scale up the *Mitanin* programme in a short time period. Special mention of the *Adhivasi Adhikar Samiti*.	Civil society mobilised action to create awareness about the PDS and provided insight.
Right to Food Campaign	Mobilised action to create awareness about nutrition and support the scale up of the *Mitanin* programme.	Mobilised action to build consumer demand and policy dialogue.
Community	Both government and non-government stakeholders have been supportive of the role of community in implementing health and nutrition programmes (several successful innovations have involved the community).	Community involved to raise awareness about PDS.
Development partners	European Union provided initial support for the *Mitanin* program in 2000. UNICEF provided technical support to ICDS since 2005.	
**Monitoring and evaluation**		
Monitoring, learning and evaluation	Data and evaluations carried out by external parties used to inform implementation of programmes.	Several social audits led by civil society on PDS used to improve implementation.

Sources: Based on perceptions of stakeholder interviews at the state level.

ICDS, Integrated Child Development Services, a programme which provides health and nutrition services to pregnant and lactating women, children below 6 years, and adolescent girls through a network of village-level centres; NGO, non-government organisation; NRHM, National Rural Health Mission was launched in 2005 to provide quality health services torural poor with a specific focus on maternal and child health, and provided additional resources to states; PDS, Public Distribution System, India’s food subsidy programme.

Our study identifies improvements in HAZ among children aged 6–59 months but not among children below 6 months of age. This is likely because stunting changes are often most visible/discernible among older children because linear growth retardation accumulates over the first year.^35^ The lack of change over time in stunting in the first few months is likely related to the fact that it is in the older infants/young children that we find the cumulative impact of the range of programmes to improve conditions during pregnancy and the first 2 years of life. To achieve improvements in growth in very young infants will require efforts to improve outcomes such as low birth weight, which have been slow to change in India.^36^ This likely requires efforts to target structural drivers such as school attendance by girls, adolescent nutrition, delaying marriage and birth spacing, which are often difficult to change in a short period.

The regression decomposition results explain 66% of the change in stunting and 81% change in HAZ over time. The proportion of change explained by the explanatory variables was nearly identical for HAZ and stunting, confirming that the subsequent analyses of policies and programmes we chose to focus on are salient.

Food, health and sanitation initiatives address the immediate determinants related to maternal and early childhood care and thus contribute to reductions in stunting. Our results are consistent with studies in Nepal^15^ and Bangladesh,^37^ which also showed health services and access to improved sanitation contributed to declines in stunting.^15 37^

Changes in health and nutrition services contributed to almost a fifth of the declines in stunting over time, affirming what the Lancet Nutrition Series has noted as the potential contribution of interventions delivered by health services. In Chhattisgarh, the *Mitanins* were envisioned to be agents of social change along with delivering community health services (table 5). Much of the improvements in infant mortality and use of health services coincided with the duration of the *Mitanin* programme.^21^ This innovation remained immune to changes in state-level political leadership due to its unique governance, maintenance of balanced political affiliations and forging of linkages with locally relevant agendas for marginalised communities.^22^

Tackling undernutrition comprehensively requires that multiple interventions or programmes reach key target households together. Improvements in SES and related outcomes such as maternal BMI contributed to 15% of change in stunting. The poverty declines, at least partly enabled by the PDS expansion, could have contributed to stunting reduction by reaching poor households with food security support. In Chhattisgarh, the implicit transfers through a well-functioning PDS had a substantial impact on rural poverty, reducing it by 39% as measured by the poverty gap index.^38^ When coupled with the increases in coverage of health and nutrition programmes, the poverty declines and sanitation improvements could also have become more ‘nutrition-sensitive’ by putting in place a set of conditions that enabled greater stunting declines than if any of these had occurred in isolation. Insights from stakeholder interviews and the literature review do not indicate that this potential ‘co-location’ occurred by design; rather it more likely occurred by default as the same areas likely saw improvements in health, ICDS, PDS and sanitation programmes. In countries like Thailand, Brazil, Peru and Mexico, similar efforts to improve a range of social determinants—poverty, sanitation, health, education—had a range of positive benefits for childhood stunting.^39^

An enabling political environment is central to achieving changes in the determinants of child undernutrition. Globally, the release of the 2008 Lancet Series on Maternal and Child Undernutrition and Scaling Up Nutrition movement facilitated to rebuild momentum for improving nutrition.^1^ In Chhattisgarh, our qualitative research highlighted that rather than the push to improve stunting, it was the need to improve the state’s ranking on development indicators that helped create a vision of impact. The state’s low ranking on poverty and infant mortality motivated both policy change and political investments. In addition, the formation of a new state provided a unique policy space and political energy (table 5). Similarly, in Odisha, high infant mortality rate acted as an impetus for action,^9^ whereas in Guatemala, Bolivia and Brazil, policy actions were driven by the need to eliminate hunger.^1^ Overall, the need for a ‘hook’ that creates a political imperative, and thus, political support, is key to success.

One of the limitations of our study is that we have not examined issues pertaining to interdistrict variability in nutrition outcomes, in implementation of programmes and the factors driving those differences. The district-level variability in the outcomes^4^ and in wealth disparities suggests the need for targeting and identifying district-based strategies.^5^ Further investigation of such differences is critical, particularly in states like Chhattisgarh where 14 districts have been identified as affected by anti-state extremism. Such districts are prone to violence, which has implications for development. In addition, we have not focused on marginalised groups in the state, which could shine a different light on the state development story. Further research is needed to deepen our understanding of substate level variability in nutritional outcomes, reasons for gaps in programme coverage, such that targeted solutions can be identified. Finally, our findings from the stakeholder interviews are limited to the perceptions of the select few who were interviewed. However, the in-depth knowledge and experience of the stakeholders are supported by the literature, lending credibility to our findings.

Our study suggests that subnational regions are a microcosm of a global scenario. It also demonstrates how regions can set their own goals and how different actors, leadership and governance can play an important role in implementation while operating under a national framework. In India, the launch of the National Nutrition Mission in 2018 provides a strong policy impetus to achieve nutrition goals. States across India can, and should, leverage this national level push on nutrition to identify mechanisms to improve implementation of nutrition-focused programmes and to reshape the social determinants of nutrition to support accelerated changes in stunting and other nutrition outcomes. Similarly, countries operating under highly devolved administration (eg, Nepal, Pakistan, Indonesia) can benefit from learnings from subnational studies on how to leverage local collaborations, develop locally relevant innovations and build on local leadership interests to achieve improvements in child health outcomes.

In conclusion, tackling undernutrition requires deliberate efforts to create policy spaces that support the implementation of policy solutions that help to create the household-level conditions that support good child growth and nutrition. Our findings suggest not only that this *is* possible, but also that it is critical for other states in India—especially those with high burdens of maternal and child undernutrition—to step up to the challenge and accelerate actions that help improve the multiple sectoral determinants of undernutrition.
