# Supplementary material for: The role of the state government, civil society and programmes across sectors in stunting reduction in Chhattisgarh, India, 2006–2016
Source: BMJ Glob Health. 2020 Jul 6;5(7):e002274. doi: 10.1136/bmjgh-2019-002274 (PMC7342433; doi:10.1136/bmjgh-2019-002274)
Supplement: Supplementary data [file bmjgh-2019-002274supp001.pdf]

Supplemental Table 1A. Stakeholder interview key questions

What do you think has changed in Chhattisgarh in the last 15 years since its inception in 2000? Do you think the lives of people have improved? How do you think that the lives of people were affected? In what ways? [Probe for what it was like when they were there]
What do you think has changed in the nutrition landscape since 2000 (*Refer to the timeline and at the end ask for feedback on the timeline)*
How has the engagement of different stakeholders such as government, civil society organizations, NGOs and Development Partners changed in the state over the years? And how has that worked for nutrition in the state?
What has been the government's role in enabling these changes that have taken place in the state? Can the changes be attributed to any specific actions taken by the government?
What do you think about the unique social fabric of Chhattisgarh (the tribal population and naxal history)- has that changed over the years and how does that relate to the above-mentioned changes?

Supplemental Table 1B. Stakeholder interview analysis code list

Category	Code List
Program information	ICDS, NRHM/NHM, PDS, NREGA
State government factors	Creation of a new state; political leadership; political stability; other political push/factors; bureaucratic leadership; other bureaucratic push/factors, state led innovation
Other stakeholder factors	Central government, Civil Society/NGOs presence/academia, individual champions, development partner presence
Facilitators	Evaluations/assessments/data, infrastructure, technology, camaraderie across stakeholders, financial factors
Impediments	High burden of IMR, mining, neglect of remote areas, Naxalism

Notes: ICDS-Integrated Child Development Services; NRHM – National Rural Health Mission; NHM – Nataionl Health Mission; PDS – Public Distribution System; NREGA – Natiaonl Rural Employment Guarantee Act

Supplemental Table 1C. Framework for analysis: Key elements for scaling up impact on nutrition (Gillespie et al 2015)

Element	Description
Vision/goal	A clear idea of what the impact would look like, accompanied by appropriate metrics and a compelling narrative that shows why it is important and how it can be achieved.
What is being scaled?	A particular intervention that is to be scaled, whether it is technology, a process, a project, innovation, and/or methodology.
Context/enabling environment	Policy/political context, implementation context such as the dedication of staff and household/community context such as social environment

Drivers and barriers	The role of stakeholders, leaders, or champions; national and local ownership, commitment creation through use of data, advocacy coalitions, and external pressures
Scaling-up strategy, processes, pathways	The type of pathways and processes considered to be appropriate, depending on the need, context, and intervention being scaled up. These include expansion of program size, type of activities, organizational strength and capacity and increase in political power
Capacity	Capacity of individuals and single organizations, systemic capacity for strategic and operational purposes.
Governance	Horizontal (cross-sectoral) and vertical (national to community) coherence, anticipating and resolving trade-offs, and commitment.
Financing	Financing of nutrition-specific and nutrition-sensitive interventions, stability of funding for building capacity; and flexibility of funding for adaptive management decisions, innovations, and learning.
Monitoring and evaluation, learning, accountability	Generating evidence of learning, and impact, expanding investments from coverage monitoring to strengthen implementation research in support of scaling-up.

Supplemental Table 2. Associations between selected factors and stunting among children 0-5.9 months in India

	2006		2016		Pooled	
	β	95% CI	β	95% CI	β	95% CI
**Immediate determinants**						
Maternal low BMI (<18.5)	0.05**	0.01,0.09	0.04***	0.02,0.06	0.04***	0.02,0.06
Vegetarian	-0.02	-0.06,0.02	0.01	-0.01,0.03	0.00	-0.01,0.02
**Health and nutrition services**						
Health and nutrition services	0.01	-0.01,0.02	0.00	-0.00,0.01	0.00	-0.00,0.01
**Underlying determinants**						
Household level						
Household size	0.00	-0.00,0.01	0.00	-0.01,0.00	0.00	-0.00,0.00
SES quintile 1	0.06	-0.02,0.14	0.02	-0.01,0.06	0.03	-0.00,0.06
SES quintile 2	0.05	-0.03,0.12	0.01	-0.02,0.04	0.01	-0.02,0.04
SES quintile 3	0.07	-0.00,0.15	0.00	-0.03,0.03	0.01	-0.02,0.04
SES quintile 4	0.00	-0.07,0.07	-0.00	-0.03,0.02	-0.00	-0.03,0.02
Any household member has health insurance	-0.00	-0.09,0.09	0.02	-0.00,0.04	0.01	-0.00,0.03
Scheduled castes	0.04	-0.02,0.09	0.02	-0.01,0.05	0.02	-0.00,0.05
Scheduled tribe	0.03	-0.04,0.10	0.04*	0.01,0.07	0.04**	0.01,0.07
Other backward classes	0.00	-0.04,0.05	0.00	-0.02,0.03	0.00	-0.02,0.02
Hindu religion	-0.02	-0.11,0.06	0.01	-0.03,0.05	0.00	-0.03,0.04
Muslim religion	-0.02	-0.11,0.07	0.02	-0.03,0.06	0.01	-0.03,0.05
Hygiene and sanitation						
Hygiene score	-0.02	-0.05,0.00	-0.01**	-0.02,-0.00	-0.01**	-0.02,-0.01
Maternal level						
High school (10-12)	0.01	-0.04,0.07	-0.03**	-0.05,-0.01	-0.02*	-0.04,-0.00
College or higher	0.08	-0.00,0.16	-0.04**	-0.07,-0.01	-0.03*	-0.06,-0.00
Married before 18	0.01	-0.03,0.05	-0.02*	-0.03,-0.00	-0.01	-0.03,0.00
**Village factors**						
% households having toilet	0.05	-0.02,0.11	0.01	-0.02,0.04	0.02	-0.01,0.04
% households having electricity	0.02	-0.04,0.07	-0.02	-0.05,0.01	-0.01	-0.04,0.01
Rural	0.01	-0.04,0.06	-0.01	-0.03,0.01	-0.01	-0.03,0.01
**Child**						
Birth order	0.00	-0.01,0.01	-0.00	-0.01,0.00	-0.00	-0.01,0.00
Boy	-0.02	-0.05,0.01	0.03***	0.02,0.05	0.02***	0.01,0.04

* $p < 0.05$, ** $p < 0.01$, *** $p < 0.001$
