# Supplementary material for: The role of the state government, civil society and programmes across sectors in stunting reduction in Chhattisgarh, India, 2006–2016
Source: BMJ Glob Health. 2020 Jul 6;5(7):e002274. doi: 10.1136/bmjgh-2019-002274 (PMC7342433; doi:10.1136/bmjgh-2019-002274)
Supplement: Supplementary data [file bmjgh-2019-002274supp002.pdf]

Supplemental Figure 1. Factors contributing to the changes in height-for-age z-score (HAZ) in Chhattisgarh between 2006-2016[1]

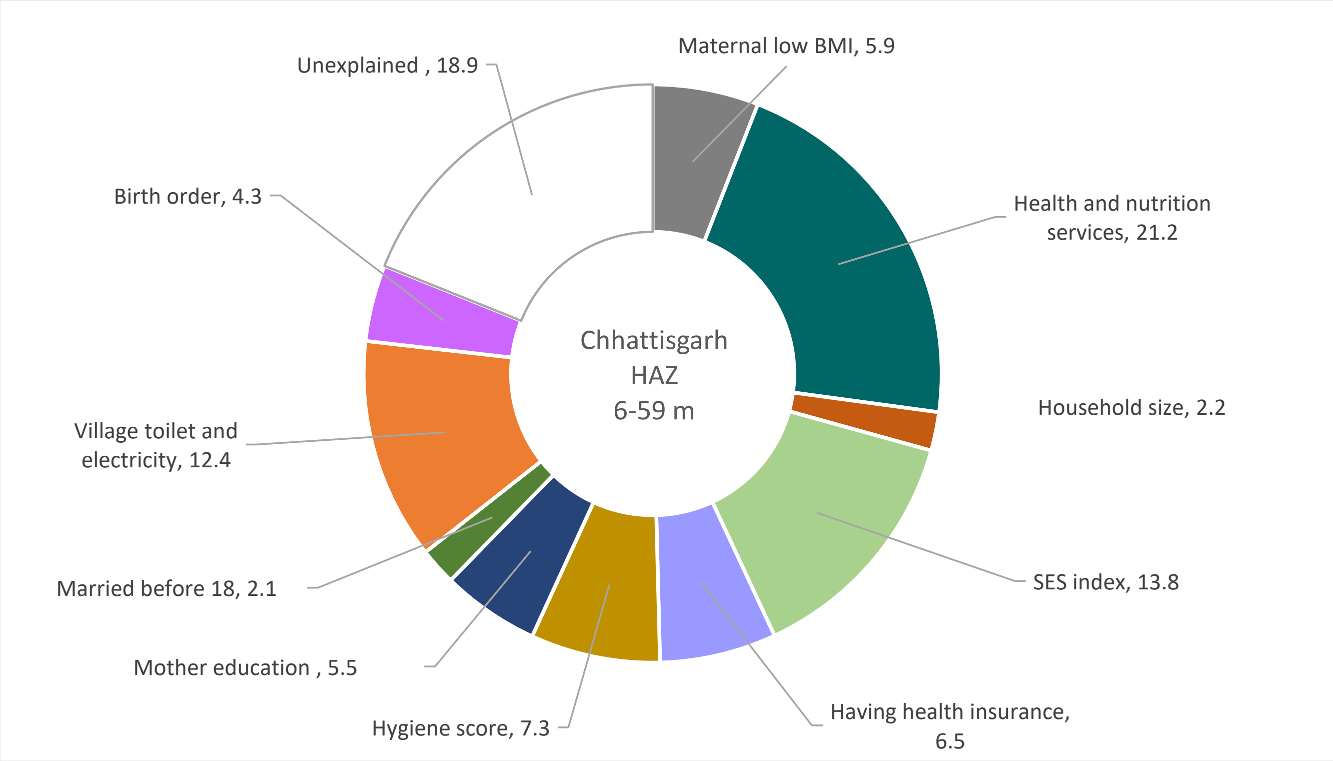

Notes: [1]Values are percent shares of the change
